# Metabolic engineering of microorganisms for the production of L-arginine and its derivatives

**DOI:** 10.1186/s12934-014-0166-4

**Published:** 2014-12-03

**Authors:** Jae Ho Shin, Sang Yup Lee

**Affiliations:** Metabolic and Biomolecular Engineering National Research Laboratory, Department of Chemical and Biomolecular Engineering (BK21 Plus Program), Center for Systems and Synthetic Biotechnology, Institute for the BioCentury, KAIST, 291 Daehak-ro, Yuseong-gu, Daejeon 305-701 Republic of Korea; BioProcess Engineering Research Center, KAIST, Daejeon, 305-701 Republic of Korea; BioInformatics Research Center, KAIST, Daejeon, 305-701 Republic of Korea

**Keywords:** Metabolic engineering, L-Arginine, L-Ornithine, Putrescine, Biopolymers, Polyaspartate

## Abstract

L-arginine (ARG) is an important amino acid for both medicinal and industrial applications. For almost six decades, the research has been going on for its improved industrial level production using different microorganisms. While the initial approaches involved random mutagenesis for increased tolerance to ARG and consequently higher ARG titer, it is laborious and often leads to unwanted phenotypes, such as retarded growth. Discovery of L-glutamate (GLU) overproducing strains and using them as base strains for ARG production led to improved ARG production titer. Continued effort to unveil molecular mechanisms led to the accumulation of detailed knowledge on amino acid metabolism, which has contributed to better understanding of ARG biosynthesis and its regulation. Moreover, systems metabolic engineering now enables scientists and engineers to efficiently construct genetically defined microorganisms for ARG overproduction in a more rational and system-wide manner. Despite such effort, ARG biosynthesis is still not fully understood and many of the genes in the pathway are mislabeled. Here, we review the major metabolic pathways and its regulation involved in ARG biosynthesis in different prokaryotes including recent discoveries. Also, various strategies for metabolic engineering of bacteria for the overproduction of ARG are described. Furthermore, metabolic engineering approaches for producing ARG derivatives such as L-ornithine (ORN), putrescine and cyanophycin are described. ORN is used in medical applications, while putrescine can be used as a bio-based precursor for the synthesis of nylon-4,6 and nylon-4,10. Cyanophycin is also an important compound for the production of polyaspartate, another important bio-based polymer. Strategies outlined here will serve as a general guideline for rationally designing of cell-factories for overproduction of ARG and related compounds that are industrially valuable.

**Electronic supplementary material:**

The online version of this article (doi:10.1186/s12934-014-0166-4) contains supplementary material, which is available to authorized users.

## Introduction

L-arginine (ARG) is a semi-essential amino acid that is important for medicinal and industrial applications. ARG is known to stimulate secretion of growth hormones [1], prolactin [2], insulin [3] and glucagon [4], promote muscle mass [5], enhance wound healing [6] and as a precursor for nitric oxide [7]. Physiological importance of ARG supplementation is further raised by the important roles of nitric oxide in cardiovascular and neurological systems [8]. For many important applications of ARG, its industrial level production has become an important task. It can be produced by microbial fermentation at an industrial scale [9] as for other amino acids such as L-glutamate (GLU) [10], L-lysine (LYS) [11], L-tryptophan (TRP) [12], L-valine (VAL) [13], L-threonine (THR) [14] and L-alanine (ALA) [15]. For these amino acids, model organisms such as *Corynebacterium glutamicum* [16] and *Escherichia coli* [17] have been widely used as production hosts, while ARG production has been performed using *B. subtilis* [18] and *C. glutamicum* [9]. It has been almost six decades since ARG production has been explored and studied using microorganisms. As in the cases for other amino acid production, random mutagenesis has been used in order to obtain efficient producer strains [19]. However, random mutagenesis is problematic due to the unwanted genomic changes introduced. Thus, much effort has been exerted to develop strains through metabolic engineering.

Systems metabolic engineering now allows construction of efficiently performing cell-factories for the microbial production of not only amino acids but also bio-fuels [20],[21], pharmaceuticals [22], bio-plastics [23], platform chemicals [24]-[26] and even silk proteins [27]. It is powered by rapidly advancing tools and continuously accumulating genetic and molecular information. It also aims to develop strains based on optimization of the entire bioprocess from strain design to industrial level cultivation. Its strategies include deletion of competing pathways [28], strengthening upstream pathways for increasing precursor pool [11], engineering transporters [29] and fine-tuning expression levels [30]. Systems metabolic engineering approach has been successfully applied in order to rationally design ARG producer strain for the efficient industrial level production which can be potentially engineered to produce ARG derivatives as well [9].

Systems metabolic engineering strategies can also be used for producing ARG-related compounds, such as L-ornithine (ORN), putrescine, and cyanophycin that share common pathways. ORN is a non-proteinogenic amino acid that has shown to improve athletic performance along with ARG and L-citrulline (CIT), another intermediate metabolite in the ARG biosynthetic pathway [31]. Putrescine is a four-carbon diamine platform chemical that can be incorporated into various polymers such as nylon-4,6 and nylon-4,10. Cyanophycin can be used to produce polyaspartate which is another bio-polymer for various technical applications. However, efficient metabolic engineering for such compounds has been limited by incomplete understanding on ARG biosynthesis even with the publically available genome sequences [32]. Here, we review the three major pathways for ARG biosynthesis in prokaryotes including the recent discoveries. We also discuss various strategies applied to engineer strains for the efficient production of ARG, ORN, putrescine and cyanophycin using recently established examples.

### L-Arginine biosynthetic pathway and its regulation

**Figure 1:**
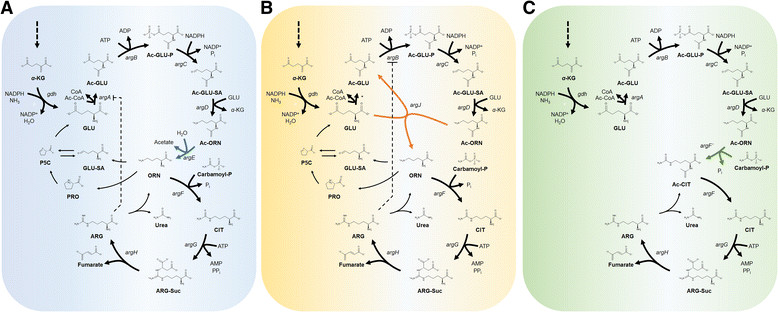
**Representative ARG biosynthesis routes in prokaryotes. (A)** The linear pathway, **(B)** the recycling pathway and **(C)** the newly discovered pathway for the ARG biosynthesis are shown. Dashed line indicates feedback inhibition by ARG on the first (NAGS) **(A)** and second (NAKG) **(B)** committed steps in the pathways. Blue arrows indicate *argE* used in the linear pathway **(A)**. Orange arrows indicate *argJ* used in the recycling pathway **(B)**. Green arrow indicates *argF’* used in the newly found pathway **(C)**. ARG catabolic pathways are also shown. Ac-GLU, *N*-acetylglutamate; Ac-GLU-P, *N*-acetylglutamyl-5-phosphate; Ac-GLU-SA, *N*-acetylglutmate-5-semialdehyde; ARG-Suc, L-argininosuccinate; GLU-SA, L-glutamate-5-semialdehyde; P5C, 1-pyrroline-5-carboxylate. The asterisk indicates putative NAGS that has not been characterized in many organisms.

In prokaryotes, there are three major biosynthetic pathways for ARG; “linear”, “recycling” and the “new” pathways (Figure 1) [33],[34]. Each pathway is comprised of eight enzymatic steps from GLU and the major differences in these pathways are in that different genes are involved for conversion of *N*-acetylornithine (Ac-ORN) for further downstream reactions toward ARG [35]. In the linear pathway (Figure 1A), Ac-ORN is converted to ORN by acetylornithinase (AOase; encoded by *argE*) [36], whereas in the recycling pathway (Figure 1B) this is catalyzed by a different enzyme, ornithine acetyltransferase (OATase; encoded by *argJ*) [37]. In the third pathway, which has not been named, ORN is bypassed and instead *N*-acetylcitrulline (Ac-CIT) is formed by acetylornithine carbamoyltransferase (AOTCase; encoded by *argF’*, Figure 1C) [38]. While certain aspects of the pathway components are still under debate, they are undoubtedly important in ARG biosynthesis and metabolic engineering purposes.

In the linear pathway (Figure 1A), GLU is converted to acetylglutamate (Ac-GLU) by *N*-acetylglutamate synthase (NAGS, encoded by *argA*) which is inhibited by ARG through negative feedback regulation [36],[39]. Sequential catalytic reactions catalyzed by the next three enzymes, *N*-acetylglutamate kinase (NAGK, encoded by *argB*), *N*-acetylglutamate semialdehyde dehydrogenase (encoded by *argC*) and *N*-acetylornithine transaminase (encoded by *argD*), which are common in the three pathways (Figure 1), yield *N*-acetylornithine (Ac-ORN) [34]. The next step, which distinguishes the linear pathway from the other two pathways, is deacetylation of Ac-ORN by AOase to yield ORN [40],[41]. The next and final steps are carried out by ornithine carbamoyltransferase (OTC or OTCase, encoded by *argF*), argininosuccinate synthase (encoded by *argG*) and argininosuccinate lyase (encoded by *argH*), which finally yield ARG [35]. This pathway has been found in a few species such as *Myxococcus xanthus* [41] and *E. coli* [36].

In many other prokaryotes including *Geobacillus stearothermophilus* (formerly *Bacillus stearothermophilus*) [37],[42],[43], *Thermotoga neapolitana* [42], Pseudomonads [44], *Neisseria gonorrhoeae* [45] *Streptomyces coelicolor* [46] and *C. glutamicum* (formerly *Micrococcus glutamicus*) [19],[47], ARG is synthesized *via* the recycling pathway and many aspects remain unknown herein (Figure 1B). The recycling pathway is regarded as more evolved and economical than the linear pathway and is “recycling” in the sense that the acetyl group deacetylated from Ac-ORN in the fifth biosynthetic step (similarly as in AOase) is re-used to acetylate GLU in the first committed step (similarly as in NAGS) of the pathway (Figure 1B). The OATase involved in the recycling step is either monofunctional or bifunctional depending on the species. For example, the OATase from *G. stearothermophilus* [37] and *N. gonorrhoeae* [40] is bifunctional and accepts both Ac-CoA and Ac-ORN as substrates to acetylate GLU, whereas that from *S. coelicolor* only accepts Ac-ORN as a substrate and considered monofunctional [46]. However, many of monofunctional OATases are mislabeled as bifunctional and some are still being corrected [48]. For example, the OATase from *C. glutamicum* which had been known to be bifunctional for decades [19],[47],[49]-[51] has been re-considered as monofunctional [52]-[54], while that from *C. crenatum* remains bifunctional [34]. For species such as *S. coelicolor*, the OATase is characterized. However, NAGS has not been identified in this bacterium, while new classes of NAGS are continuously being discovered for other species [53]. For example, the novel type of NAGS (C-NAGS) [53] encoded by *cg3035* from *C. glutamicum* adds to the diversity of NAGS including (1) the classical NAGS (as in the linear pathway), (2) the bifunctional OATase (as in the recycling pathway), (3) ArgH(A) fusion types (*argH*-*argA* fusion) [55], and (4) the short versions of NAGS (S-NAGS) [56]. Additionally, for species that have both NAGS and OATase such as *G. stearothermophilus* [43] and *N. gonorrhoeae* [57], there is a functional redundancy and the NAGS function is regarded as anaplerotic to replenish Ac-GLU [57],[58]. Moreover, another distinctive feature of this pathway is that NAGK reaction instead of NAGS reaction is negatively regulated by ARG [44],[52],[59],[60].

In the newly discovered pathway (Figure 1C), AOTCase from *Xanthomonas campestris* transfers carbamoyl group from carbamoyl phosphate to Ac-ORN to form Ac-CIT [38]. Here, the formation of ORN is bypassed and ArgE deacetylates Ac-CIT to yield CIT. While the details of this pathway, as with the linear and recycling pathways, have not been fully explored, *C. glutamicum* and its related species with the recycling pathway are recognized as the organisms to most efficiently produce ARG.

In terms of the chromosomal genetic organization, ARG biosynthetic genes are diversely organized in different species, and that from *C. glutamicum* has been studied the most. In *C. glutamicum*, the *argCJBDFRGH* cluster is organized into two operons (*argCJBDFR* and *argGH*) [52] and transcription of these operons are regulated by ARG [61], ArgR [62] and FarR [63], while the putative *argA* (*cg3035*, encoding C-NAGS) is separated from this cluster [32],[52],[53]. FarR regulates transcription of the *arg* operon by binding to the upstream of *argC*, *argB*, *argF* and *argG* genes [63],[64]. FarR additionally controls the ARG biosynthesis by binding to the upstream of the *gdh* gene encoding glutamate dehydrogenase which converts *α*-ketoglutarate (*α*-KG) into GLU [63]. Similarly, ArgR, a global regulator, binds to *argC* and *argG* promoters to control ARG biosynthesis [49] and the degree of down-regulation is increased by ARG [61] but its binding affinity decreases by L-proline (PRO), which can be considered as a stimulator for ARG biosynthesis [65]. Additionally, other strains have different chromosomal organization in the ARG operon. For example, it is partially clustered in the order of *argCJBD* in the chromosome for gram-positive bacteria such as *G. stearothermophilus* and *S. coelicolor* [46],[66], while the bipolar organization of *argECBH* is found in gram-negative bacteria such as *E. coli* [67]-[70].

### Metabolic engineering for L-arginine production

Initial approach to produce ARG at industrial scale began with random mutagenesis of microorganisms (Table 1). Mutants selected based on their resistance to antimetabolites and other analogues such as canavanine (CVN) [40],[71], homoarginine [72], arginine hydroxamate (AHX) [18],[73], 6-azauracil (6 AU) [74], 2-thiazolealanine (TA) [75], and sulfaguanine (SG) [75] have been used in early attempts to overproduce ARG. Mutations were induced by radiation [75],[76] or treatment with mutagen such as *N*-methyl-*N*’-nitro-*N*-nitrosoguanidine (NTG) [18],[75]. The rational for this is to confer higher tolerance of ARG to microorganisms and to remove feedback inhibition by ARG [9]. Historically, the random mutation approach had been used in various prokaryotic and eukaryotic strains including *B. subtilis* [18],[73],[74], *Serratia marcescens* [73], *Micrococcus sodonensis* [73], *Norcadia corynebacteroides* [73], *N. rubra* [73], *Saccharomyces cerevisiae* [73], *Candida tropicalis* [73]*, C. glutamicum* [72], *C. crenatum* [76], *Brevibacterium flavum* [75], *B. ketoglutamicum* [75], *C. lilium* [75], *Arthrobacter paraffineus* [75] and *Microbacterium ammoniaphilum* [73],[75] to produce ARG. The trend later shifted toward using GLU overproducing *C. glutamicum* strain and its related species *C. crenatum* as base strains, which led to industrial level ARG titers. More importantly, the random mutation method is now used in synergistic combination with high-throughput molecular tools which enables systems metabolic engineering for industrial microbial strain development.

**Table 1:** **ARG, ORN, putrescine and cyanophycin producing strains**

Product	Year	Strain (vector if any)	Remark	Titer (g/liter)	Reference
ARG	1971	AHr-5	AHX resistant *B. subtilis*; test tube culture	4.5	[18]
	1973	AJ 3351	*B. ketoglutamicum* ATCC 15587 mutant; SG^R^,	2.1	[75]
		AJ 3352	*A. paraffineus* ATCC 19065 mutant; TA^R^,	1.2	
		AJ 3353	*M. ammoniaphilum* ATCC 15354 mutant; TA^R^,	2.9	
		No. 348	*C. lilium* NRRL B-2243 mutant; TA^R^	1.8	
		No. 352	*B. flavum* ATCC 14067 mutant; guanine auxotroph; TA^R^	34.8	
	1977	AAr-9	*B. subtilis* OUT 8103 mutant; 6AU^R^	28.0	[74]
	1981	KY7690	*B. subtilis* ATCC 15244 mutant; AHX^R^, 5HUR^R^, TRA^R^, 6FTP^R^, 6AU^R^, 2TU^R^; 5 liter bioreactor	14.0	[73]
		*S. marcescens*	IFO 3046 mutant; AHX^R^, NIM^R^; test tube culture	0.6	
		*M. ammoniaphilum*	ATCC 15354 mutant; AHX^R^; test tube culture	0.5	
		*M. sodonensis*	ATCC 11880 mutant; AHX^R^; test tube culture	4.0	
		*N. corynebacteroides*	ATCC 14898 mutant; CVN^R^; test tube culture	2.5	
		*N. rubra*	NRRL 11094 mutant; AHX^R^; test tube culture	8.0	
	2009	RBid	*C. glutamicum* ATCC 13032, *ΔargR*, A26V/M31V in ArgB; 5 liter bioreactor	52.0	[47]
	2009	SYPA 5-5	*C. crenatum* mutant; optimization of two-stage oxygen supply strategy; 5 liter bioreactor	36.6	[76]
	2011	SYPA 5–5 (pJC-*tac*-*vgb*)	*C. crenatum* mutant, vector-based overexpression of *vgb* from *Vitreoscilla*	35.9	[77]
	2011	SYPA 5–5 (pJC*tac*-*argJ*)	*C. crenatum* mutant, vector-based overexpression of *argJ*; 5 liter bioreactor	42.4	[34]
	2012	SYPA-9039 (pJC-9039)	*C. crenatum* SYPA5-5 harboring with vector-based overexpression of *argCJBDFRGH*; 5 liter bioreactor	45.3	[78]
	2014	AR6	*C. glutamicum* ATCC 21831; AHX^R^, CVN^R^, *ΔargR*, *ΔfarR*, *pgi* (A1G), *Psod::tkt, tal, zwf, opcA, pgl, ΔNCgl1221*, *Psod::carAB*, *Petfu::argGH*; 5 liter bioreactor	92.5	[9]
			Same as above except 1,500 liter bioreactor	81.2	
ORN	1996	BK533	*B. ketoglutamicum* ATCC 21092 derived mutant; UV and NTG treatment; 2 liter bioreactor	5.7	[79]
	2008	SJ8074 (pEK-CJBD)	*C. glutamicum* ATCC 13032 *ΔargF*, *ΔargR, ΔproB,* vector-based overexpression of *argCJBD*	0.179	[80]
	2010	SJ8074 (pEK-P_trc_::1469)	*C. glutamicum* ATCC 13032 *ΔargF*, *ΔargR, ΔproB,* vector-based overexpression of *NCgl1469*	0.320	[54]
	2010	*C. glutamicum*	ATCC 13032; proline supplement	3.295	[62]
	2011	ORN1 (pVWEx1-*araBAD*)	*C. glutamicum* ATCC 13032 *ΔargF*, *ΔargR*, vector-based overexpression of *araBAD*; optimal ARG supplement, arabinose supplement	25.77	[81]
	2012	*C. glutamicum*	ATCC 13032 *ΔargF*, *ΔproB, Δkgd*	4.78	[82]
	2012	SJC8399	*C. glutamicum* ATCC *ΔargF*, *ΔargR*, *ΔNcgl2399*, *ΔNcgl2905*	13.16	[83]
	2013	*Δ*APRE::*rocG*	*C. glutamicum* ATCC 13032 *ΔargF*, *ΔproB*, *ΔargR, ΔspeE::*P_*tac*-*M*_-*rocG*	14.84	[84]
	2013	*Δ*APE6937R42	*C. glutamicum* ATCC 13032 *ΔargF*, *ΔproB*, *ΔspeE*, *ΔargR*; 70 passages of adaptive evolution	24.1	[85]
	2014	YW6 (pSY233)	*C. glutamicum* ATCC 13032 *ΔproB*, *ΔargF*, *ΔargR*, pgi^GTG^, zwf^ATG^, *Ptkt*::*Psod*, vector-based overexpression of *argCJBD* from *C. glutamicum* ATCC 21831; 5 liter bioreactor	51.5	[86]
Putrescine	2009	XQ52 (p15SpeC)	*E. coli* W3110 *ΔlacI, ΔspeE, ΔspeG, ΔargI, ΔpuuPA,* P*argECBH*::P*trc*, P*speF*-*potE*::P*trc,* P*argD*::P*trc,* P*speC*::P*trc*, *ΔrpoS*, vector-based overexpression of *speC*; 5 liter bioreactor	24.2	[28]
	2010	ORN1 (pVWEx1-*speC*)	*C. glutamicum* ATCC 13032 *ΔargF*, *ΔargR*, vector-based overexpression of *speC* from *E. coli* MG1655; ARG supplement,	6.0	[50]
	2012	ORN1 (pVWEx1-*speC*-*5*’_*21*_-*argF*)	*C. glutamicum* ATCC 13032 *ΔargF*, *ΔargR*, vector-based overexpression of *speC* from *E. coli* MG1655; ARG auxotrophy rescue by fine-tuned *argF* expression *via* plasmid-addiction system	19.0	[51]
	2013	ORN1 (pVWEx1-*speC*-*5*’_*21*_-*argF*)	*C. glutamicum* ATCC 13032 *ΔargF*, *ΔargR*, vector-based overexpression of *speC* from *E. coli* MG1655; glycerol and glucose as carbon source; ARG supplement	0.855	[87]
Cyanophycin	2001	*C. glutamicum* (pEK0::*cphA*)	ATCC 13032, vector-based overexpression of *cphA* from *Synechocystis* sp. Strain PCC6308	3.6	[88]
		H16-PHB^−^4 (pBBR1::*cphA*)	*R. eutropha* DSM 541 (DSM 428 derivative), PHA synthesis defect, vector-based overexpression of *cphA* from *Synechocystis* sp. Strain PCC6308	8.7	
		*E. coli* (pSK::*cphA*)	TOP 10, vector-based overexpression of *cphA* from *Synechocystis* sp. Strain PCC6308	26.0	
		*P. putida* (pBBR1::*cphA*)	KT2440, vector-based overexpression of *cphA* from *Synechocystis* sp. Strain PCC6308	11.0	
	2002	*E. coli* (pMa/c5-914::*cphA*)	DH1 strain, vector-based overexpression of *cphA* from *Synechocystis* sp. PCC6803; 30 liter bioreactor	24.0	[89]
			Same as above except 500 liter bioreactor	21.0	
	2004	*E. coli* (pSK::*cphA1*_7120_)	TOP 10, vector-based overexpression of *cphA1* from *Anabaena* sp. strain PCC7120	21.0	[90]
		GPp104 (pBBR1MCS-2::*cphA1*_7120_)	*P. putida* KT2440 mutant, vector-based overexpression of *cphA1* from *Anabaena* sp. strain PCC7120	24.0	
		H16-PHB^−^4 (pBBR1MCS-2::*cphA1*_7120_)	*R. eutropha* DSM 541 (DSM 428 derivative), PHA synthesis defect, vector-based overexpression of *cphA1* from *Anabaena* sp. strain PCC7120	22.0	
	2005	*A. calcoaceticus*	ATCC 33305, flask cultivation; ARG supplement	46.0	[91]
	2006	*R. eutropha* (pBBR1MCS-2::*cphA/eda*)	DSM 541 derivative, H16-PHB^−^4 *Δeda*, vector-based overexpression of *cphA* from *Synechocystis* sp. Strain PCC6308; *eda*-dependent plasmid-addiction system; flask cultivation	40.0	[92]
			Same as above except 30 liter bioreactor	35.8	
			Same as above except 500 liter bioreactor	32.0	
	2011	*E. coli* (pCOLADuet-1::*cphA*C595S::*dapL*_Ss_)	HMS174(DE3) *ΔdapE*, plasmid-addiction system using *dapL* from *Synechocystis* sp. Strain PCC6308, C595S mutant *cphA* from *Synechocystis* sp. Strain PCC6308; flask cultivation	42.0	[93]
			Same as above except 25 liter bioreactor	14.1	
			Same as above except 400 liter bioreactor	18.0	
	2012	*R. eutropha* (pBBR1MCS-2::*cphA*_6308_ */eda*_H16_)	DSM 428 mutant, H16-PHB^−^4 *Δeda*, vector-based overexpression of *cphA* from *Synechocystis* sp. Strain PCC6308, *eda*-dependent plasmid-addiction system; 30 liter bioreactor	47.5	[94]

Strains that have been reported to produce ARG, ORN, putrescine and cyanophycin are listed in the order of year for each compound. The relevant genetic information and production titers are shown. All cyanophycin production titers are given in a different unit scale (w/w %) than the rest which are given in g/liter. 5HUR, 5-hydroxyuridine; TRA, triazolealanine; 6FTP, 6-fluorotryptophan; 2TU, 2-thiouracil; 5FU, 5-fluorouracil; NIM, polyoxyethylene stearylamine.

The strategies for rationally designing ARG overproducer typically consist of (1) removal of feedback inhibition, (2) overexpression of the biosynthetic genes (e.g., the *arg* operon) and/or removal of the repressors (e.g., *argR* and *farR*), (3) increasing NADPH pool required for ARG biosynthesis, (4) increasing carbamoyl phosphate pool by overexpression of *carAB* operon and (5) deletion of exporter for GLU encoded by *NCgl1221*. For example, reverse engineering approach was taken to the wild-type *C. glutamicum* ATCC 13032 strain for deleting *argR* and introducing A26V and M31V mutations in ArgB in order to alleviate feedback inhibition [47]. This is an important study because it presented the first genetically defined and not randomly mutated strain for ARG production and the engineered strain produced 52 g/liter of ARG [47]. Plasmid-based engineering system has also been explored. Overexpression of a bacterial hemoglobin from *Vitreoscilla* in *C. crenatum* SYPA 5*–*5 for increased dissolved oxygen availability led to the production of 35.9 g/liter ARG [77]. Plasmid-based overexpression of the *argCJBDFRGH* cluster or *argJ* alone in *C. crenatum* SYPA 5*–*5 also led to enhanced ARG production, reaching 45.3 g/liter or 42.4 g/liter, respectively [34],[78]. A possible explanation for little difference in ARG titer here despite the different number of gene overexpression is probably because different cultivation conditions were used (e.g., different temperatures).

Along the same line, a recent systems metabolic engineering study led to a very successful production of ARG at the industrial-scale [9]. *C. glutamicum* ATCC 21831 was initially treated with CVN and AHX in order to increase its ARG tolerance and subjected to stepwise strain development. The *argR* and *farR* genes were deleted in order to relieve negative regulation on ARG biosynthesis. Next, in order to improve the NADPH pool, the pentose phosphate pathway (PPP) flux was enhanced by reducing the *pgi* expression through replacing ATG start codon with GTG, and overexpressing the major PPP operon consisting the *tkt, tal, zwf, opcA* and *pgl* by replacing the native promoter with the strong *sod* promoter. Finally the promoters for *carAB* and *argGH* operons were also changed in order to optimize fluxes toward the ARG biosynthesis and the *Ncgl1221* gene*,* encoding the GLU exporter, was deleted. As a result, the final constructed strain produced 92.5 g/liter and 81.2 g/liter of ARG at the laboratory-scale and at the industrial-scale fermentations, respectively [9]. This work is a good example of systems metabolic engineering for developing a microbial strain capable of overproducing ARG to the level and performance suitable for industrial-scale production.

### Metabolic engineering for L-ornithine production

**Figure 2:**
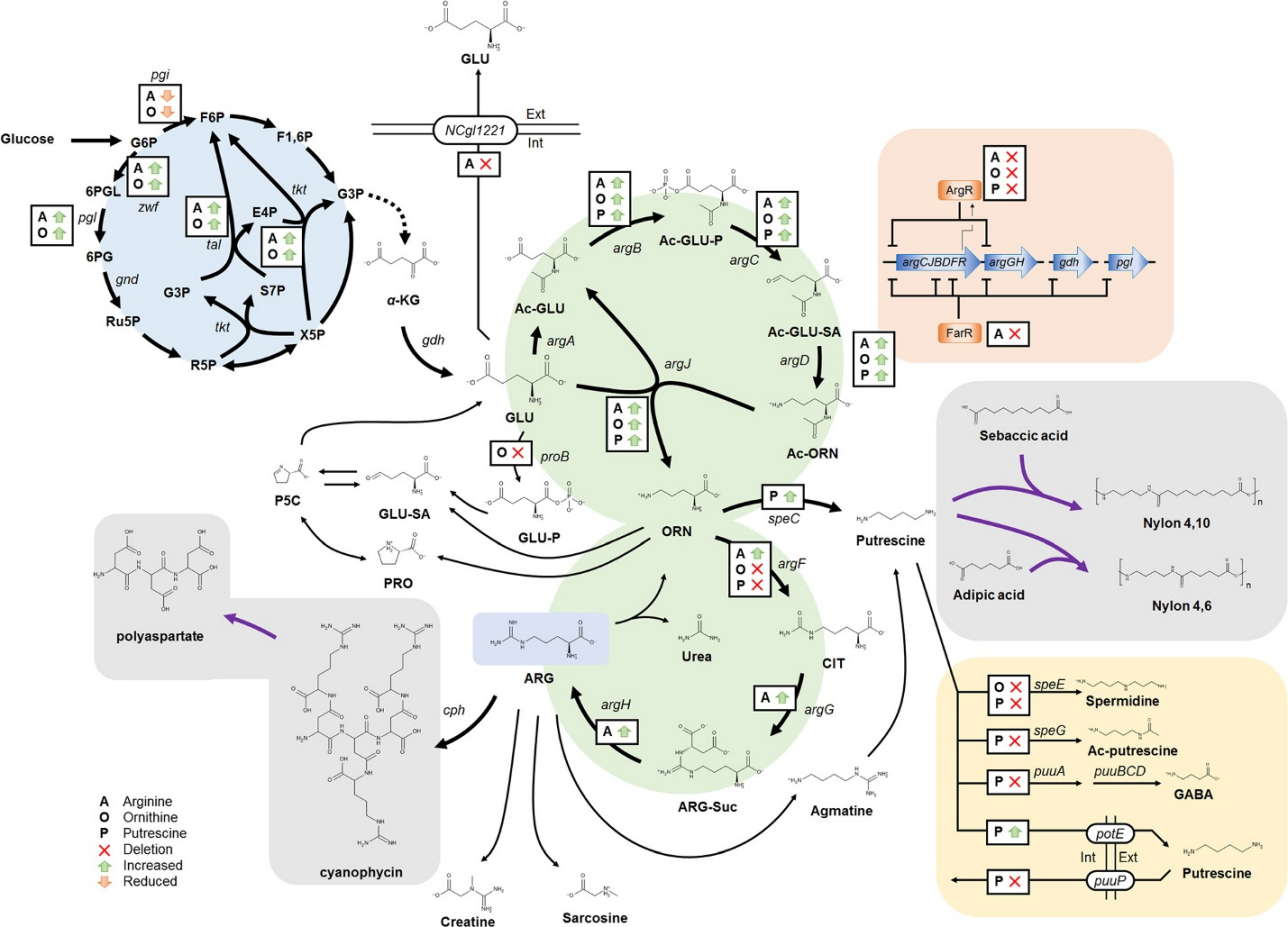
**Systems metabolic engineering strategies.** The metabolic engineering strategies used for the construction of microbial strains producing ARG, ORN, putrescine and cyanophycin are shown. Increased and decreased gene expression levels are shown in green and orange arrows. Purple arrows indicate reactions carried out by chemical means. The *pgi* encodes phosphoglucose isomerase, *zwf* encodes glucose-6-phosphate dehydrogenase, *pgl* encodes 6-phosphogluoconolactonase, *gnd* encodes 6-phosphogluconate dehydrogenase, *tkt* encodes transketolase and *tal* encodes transaldolase. Gene deletion is indicated with red crosses. GABA, gamma-aminobutyrate; Ac-putrescine, acetyl-putrescine; G6P, glucose-6-phosphate; F6P, fructose-6-phosphate; F1,6P, fructose-1,6-bisphosphate; 6PGL, 6-phosphogluconolactone; 6PG, 6-phosphogluconate; Ru5P, ribulose-5-phosphate; R5P, ribose-5-phosphate; G3P, glyceraldehyde-3-phosphate; X5P, xylulose-5-phosphate; S7P, sedoheptulose-7-phosphate; E4P, erythrose-4-phosphate; A, ARG; O, ORN; P, putrescine.

The ARG-derivative, ORN, has also been produced by microbial fermentation. Both the strategies of random mutagenesis [79] and systems metabolic engineering have been employed for developing strains (Figure 2). In rationally designing an ORN producer, knocking out the competing branches to redirect carbon flux to ORN pathway is an important and common strategy. Specifically, the strategies of PRO supplement [62], ARG supplement [81], vector-based overexpression of *argCJBD* [80], *NCgl1469* overexpression [54], overexpression of *rocG* from *B. subtilis* [84], *ΔargF* [80]-[85], *ΔproB* [82],[84],[85], *ΔspeE* [84],[85], *ΔargR* [81],[83]-[85], *ΔNCgl2399* [83]*, ΔNCgl2905* [83], and *Δkgd* [82] have been employed for developing strains for ORN overproduction (Table 1).

The strategies for the development of ORN producers are similar to those employed for ARG producers except auxotrophy rescue by supplements is additionally used. Here, *ΔargF* and *ΔproB* are often included in order to disrupt OTCase and gamma-glutamyl kinase, respectively [80]-[85]. Although this strategy leads to higher ORN titer, it makes the strain auxotrophic for ARG and PRO since their biosynthesis is disrupted [62],[81]. Another common strategies are deletion of the repressor (*ΔargR*) [81],[83]-[85] as in ARG strain cases, and overexpression of the biosynthetic genes (e.g., *argCJBD*) using plasmids [80]. Overexpression of putative biosynthetic genes can also be a strategy for ORN production. It has been reported that overexpression of putative NAGS encoded by *NCgl1469* leads to increased ORN production [54] while others claim *Ncgl1469* as diaminopentane acetyltransferase [95]. It is possible that *Ncgl1469* potentially encodes a broad-substrate acetyltransferase that has not been characterized in detail. The TCA cycle flux can also be reduced by deleting 2-oxoglutarate dehydrogenase complex (ODHC) for the enhanced production of ORN [82].

Increasing the NADPH pool also improves ORN production. The use of *B. subtilis rocG* which encodes NAD-dependent glutamate dehydrogenase allows conversion of *α*-KG to GLU in an NADPH-independent manner and leaves more NADPH for ORN biosynthesis [84]. Increasing the NADPH level can also be achieved by inactivating two putative gluconate kinases (*gntK*) encoded by *NCgl2399* and *NCgl2905* [83]. Overexpression of the ATP-dependent NAD kinase encoded by *ppnK* also leads to enhanced ORN production, while overexpression of glucose-6-phosphate dehydrogenase encoded by *zwf* and 6-phosphogluconate dehydrogenase encoded by *gnd* does not do the same [85]. A possible explanation is that plasmid-based overexpression of *zwf* and *gnd* causes cellular burdens because chromosomal-level overexpression has shown improvement in ORN titer [86]. An indirectly associated pathway for spermidine biosynthesis can also be deleted for enhanced ORN production, yet the reason behind it has not been explained [85]. Combining the aforementioned strategies, a recently developed strain was reported to produce 51.5 g/L of ORN [86]. In this strain, the PPP flux was enhanced by changing the *tkt* promoter and the start codons of *pgi* and *zwf*. The *argCJBD* cluster from *C. glutamicum* ATCC 21831 was overexpressed and *argF*, *proB* and *argR* were deleted.

### Metabolic engineering for putrescine production

Putrescine (1,4-diaminobutane) can be produced by metabolic engineering of ARG related pathways. The major chassis organisms that have been employed are *E. coli* [28] and *C. glutamicum* [50]. While the putrescine biosynthesis pathway is not well known in *C. glutamicum,* it is a desirable host as it produces ORN efficiently and tolerates putrescine better than *E. coli* [28],[50]. Although putrescine biosynthesis can be alternatively achieved *via* agmatine pathway (Figure 2), the ODC pathway was shown to be more efficient than the agmatine pathway [50]. In addition to the strategies employed for developing ARG and ORN producers described in prior sections, engineering the transporters are the additional strategies for designing cell-factories for putrescine production.

Putrescine can be synthesized from ORN by a single reaction carried out by ornithine decarboxylase (ODC) encoded by *speC* (Figure 2). The ODC from *E. coli* is often used since the metabolic pathway in *C. glutamicum* for putrescine has not been identified [50]. To develop a putrescine producing *C. glutamicum* strain, the arginine repressor encoded by *argR* should be inactivated as in the ARG and ORN overproducing strains [50],[51]. While disruption of OTCase is also a strategy for improving ORN pool, this makes the strain to become an ARG auxotroph [50]. Here, the ARG auxotrophy caused by *ΔargF* can be overcome by introducing a plasmid expressing *argF* which is also fine-tuned [51]. The use of this strategy is an example of plasmid-addiction system and it circumvents the undesirable use of antibiotic as well because cell viability becomes plasmid-dependent [96]. While engineering of the putrescine transport system in *C. glutamicum* would further enhance its production, this strategy has yet been applied only in *E. coli* [28]. Along with the overexpression of putrescine/ornithine antiporter (encoded by *potE*) and deletion of putrescine importer (encoded by *puuP*), the competitive and degradation routes were deleted in the putrescine producing *E. coli* XQ52 strain [28]. Chromosomal deletion of *puuA* encoding glutamate-putrescine ligase, *speE* encoding spermidine synthase, *speG* encoding spermidine acetyltransferase, and *argI* encoding one of the monomers for OTCase improved putrescine production. The native promoters of the key biosynthetic genes (*argECBH* operon, *argD* and *speC*) were changed to stronger promoters and the repressor *argR* was deleted. The *rpoS* gene encoding the stress-responsive RNA polymerase sigma factor was also deleted, which led to the development of the final strain capable of producing 24.2 g/liter of putrescine [28]. While the highest putrescine producing strain reported is so far *E. coli*, further engineering of ORN overproducing *C. glutamicum* strain will likely led to the development of a more efficient putrescine producer due to its high-tolerance to putrescine [86].

### Metabolic engineering for cyanophycin production

Cyanophycin was first discovered more than a century ago in cyanobacteria as a carbon and nitrogen storage compound [97]. Cyanophycin has been recently attracting attention because it can be chemically reduced to make polyaspartate. Polyaspartate is a completely biodegradable polymer [88], which can be used as a polyacrylate substitute, an additive polymer in the oil field [98], and as a polymer suitable for water treatments and medical applications [99]. Additionally, cyanophycin can also be used to produce isotope-labeled ARG [100].

Cyanophycin is composed of equimolar amount of ARG and L-aspartate (ASP). Cyanophycin synthetase encoded by *cphA* carries out the reaction of polymerizing ASP and ARG (Figure 2). Various strains including *P. putida* [88],[90], *R. eutropha* [88],[92],[94], *C. glutamicum* [88] and *E. coli* [88],[89],[93] have been employed for the production of cyanophycin through the heterologous expression of *cphA* from *Synechocystis* sp. PCC6803 [89] or *Anabaena* sp. strain PCC7120 [90]. *Acinetobacter calcoaceticus* [91],[101] has also been used to produce cyanophycin using the endogenous *cphA* gene [91],[101]. The metabolic engineering strategies employed include the use of mutants incapable of accumulating polyhydroxyalkanoates [88],[92],[94], plasmid-addiction system using *eda* [92],[94] or *dapE* [93] deleted strains, and the use of CphA variant having C595S mutation [102]. There was an interesting report on the use of 2-keto-3-deoxy-6-phosphogluconate aldolase encoded by *eda,* which is required in gluconate and fructose metabolism. The use of this gene for plasmid-addiction system in *Δeda* strain circumvents the need to use antibiotics in large-scale cultivation. The 30, 400 and 500 liter-scale bioreactors have been used for the large-scale production of cyanophycin, which was followed by successful purification; at the end, the titer corresponding to 750 g of cyanophycin with 75% extraction yield have been reported [89].

## Conclusions

With increasing volumes of biological information and availability of high-throughput molecular tools, systems metabolic engineering has become an essential strategy for developing microbial strains overproducing ARG, ORN, putrescine and cyanophycin. Systems metabolic engineering obviously requires thorough understanding of the metabolism and gene regulatory circuits towards the production of desired products. The strategies of knocking out the negative regulatory mechanisms, amplifying the fluxes of pathways towards the product formation, deleting the byproducts forming pathways, and increasing the exporters while reducing the importers have been combined to develop microbial strains capable of producing ARG and related products. Such engineering strategies have been successfully applied to rationally construct a high-performance strain which works efficiently not only at the laboratory-scale but also at the semi industrial-scale fermentation. New tools of systems metabolic engineering are continuously emerging. For example, further metabolic engineering of the strain based on the sRNA technology can be envisioned to rapidly develop high-level producers. The strategies described here will be useful for developing microbial strains capable of more efficiently producing ARG and related products, including not only those mentioned in this paper but also other derivatives including sarcosine, creatine, agmatine and creatinine.

## Authors’ original submitted files for images

Below are the links to the authors’ original submitted files for images. Authors’ original file for figure 1 Authors’ original file for figure 2

## Abbreviations

ARG: L-arginine
LYS: L-lysine
TRP: L-tryptophan
VAL: L-valine
THR: L-threonine
ALA: L-alanine
ASP: L-aspartate
ORN: L-ornithine
PRO: L-proline
CIT: L-citrulline
GLU: L-glutamate
Ac-ORN: *N*-acetylornithine
Ac-CIT: *N*-acetylcitrulline
Ac-CoA: Acetyl coenzyme A
*α*-KG: *α*-ketoglutarate
OATase: Ornithine acetyltransferase
OTCase: Ornithine cabamoyltransferase
NAGS: *N*-acetylglutamate synthase
NAGK: *N*-acetylglutamate kinase
AOase: Acetylornithinase
AOTCase: Acetylornithine carbamoyltransferase
ODC: Ornithine decarboxylase
PPP: Pentose phosphate pathway
AHX: Arginine hydroxamate
CVN: Canavanine
6 AU: 6-azauracil
TA: 2-thiazolealanine
SG: Sulfaguanine
NTG: *N*-methyl-*N*’-nitro-*N*-nitrosoguanidine
NADPH: Nicotinamide adenine dinucleotide phosphate
ATP: Adenosine triphosphate

## References

[1] Albaroth J, Muller OA, Schopohl J, Vonwerder K (1988). Arginine stimulates growth-hormone secretion by suppressing endogenous somatostatin secretion. J Clin Endocrinol Metab.

[2] Davis SL (1972). Plasma levels of prolactin, growth-hormone, and insulin in sheep following infusion of arginine, leucine and phenylalanine. Endocrinology.

[3] Thams P, Capito K (1999). L-Arginine stimulation of glucose-induced insulin secretion through membrane depolarization and independent of nitric oxide. Eur J Endocrinol.

[4] Palmer JP, Benson JW, Walter RM, Ensinck JW (1976). Arginine-stimulated acute phase of insulin and glucagon-secretion in diabetic subjects. J Clin Invest.

[5] Jobgen WJ, Meininger CJ, Jobgen SC, Li P, Lee MJ, Smith SB, Spencer TE, Fried SK, Wu GY (2009). Dietary L-arginine supplementation reduces white fat gain and enhances skeletal muscle and brown fat masses in diet-induced obese rats. J Nutr.

[6] Barbul A, Lazarou SA, Efron DT, Wasserkrug HL, Efron G (1990). Arginine enhances wound-healing and lymphocyte immune-responses in humans. Surgery.

[7] Rogers NE, Ignarro LJ (1992). Constitutive nitric-oxide synthase from cerebellum is reversibly inhibited by nitric-oxide formed from L-arginine. Biochem Biophys Res Commun.

[8] Ignarro LJ, Cirino G, Casini A, Napoli C (1999). Nitric oxide as a signaling molecule in the vascular system: an overview. J Cardiovasc Pharmacol.

[9] Park SH, Kim HU, Kim TY, Park JS, Kim SS, Lee SY (2014). Metabolic engineering of *Corynebacterium glutamicum*for L-arginine production. Nat Commun.

[10] Chen N, Du J, Liu H, Xu QY (2009). Elementary mode analysis and metabolic flux analysis of L-glutamate biosynthesis by *Corynebacterium glutamicum*. Ann Microbiol.

[11] Becker J, Zelder O, Hafner S, Schroder H, Wittmann C (2011). From zero to hero-design-based systems metabolic engineering of *Corynebacterium glutamicum*for L-lysine production. Metab Eng.

[12] Leuchtenberger W, Huthmacher K, Drauz K (2005). Biotechnological production of amino acids and derivatives: current status and prospects. Appl Microbiol Biotechnol.

[13] Park JH, Lee KH, Kim TY, Lee SY (2007). Metabolic engineering of *Escherichia coli*for the production of L-valine based on transcriptome analysis and in silico gene knockout simulation. Proc Natl Acad Sci U S A.

[14] Lee KH, Park JH, Kim TY, Kim HU, Lee SY (2007). Systems metabolic engineering of *Escherichia coli*for L-threonine production. Mol Syst Biol.

[15] Jojima T, Fujii M, Mori E, Inui M, Yukawa H (2010). Engineering of sugar metabolism of *Corynebacterium glutamicum*for production of amino acid L-alanine under oxygen deprivation. Appl Microbiol Biotechnol.

[16] Becker J, Wittmann C (2012). Bio-based production of chemicals, materials and fuels - *Corynebacterium glutamicum*as versatile cell factory. Curr Opin Biotechnol.

[17] Becker J, Wittmann C (2012). Systems and synthetic metabolic engineering for amino acid production - the heartbeat of industrial strain development. Curr Opin Biotechnol.

[18] Kisumi M, Kato J, Sugiura M, Chibata I (1971). Production of L-arginine by arginine hydroxamate-resistant mutants of *Bacillus subtilis*. Appl Microbiol.

[19] Udaka S, Kinoshita S (1958). Studies on L-ornithine fermentation I. The biosynthetic pathway of L-ornithine in *Micrococcus glutamicum*. J Gen Appl Microbiol.

[20] Lee J, Jang YS, Choi SJ, Im JA, Song H, Cho JH, Seung DY, Papoutsakis ET, Bennett GN, Lee SY (2012). Metabolic engineering of *Clostridium acetobutylicum*ATCC 824 for isopropanol-butanol-ethanol fermentation. Appl Environ Microbiol.

[21] Jang YS, Malaviya A, Lee J, Im JA, Lee SY, Lee J, Eom MH, Cho JH, Seung DY (2013). Metabolic engineering of *Clostridium acetobutylicum*for the enhanced production of isopropanol-butanol-ethanol fuel mixture. Biotechnol Prog.

[22] Paddon CJ, Westfall PJ, Pitera DJ, Benjamin K, Fisher K, McPhee D, Leavell MD, Tai A, Main A, Eng D, Polichuk DR, Teoh KH, Reed DW, Treynor T, Lenihan J, Fleck M, Bajad S, Dang G, Dengrove D, Diola D, Dorin G, Ellens KW, Fickes S, Galazzo J, Gaucher SP, Geistlinger T, Henry R, Hepp M, Horning T, Iqbal T (2013). High-level semi-synthetic production of the potent antimalarial artemisinin. Nature.

[23] Jung YK, Kim TY, Park SJ, Lee SY (2010). Metabolic engineering of *Escherichia coli*for the production of polylactic acid and its copolymers. Biotechnol Bioeng.

[24] Park SJ, Kim EY, Noh W, Park HM, Oh YH, Lee SH, Song BK, Jegal J, Lee SY (2013). Metabolic engineering of *Escherichia coli*for the production of 5-aminovalerate and glutarate as C5 platform chemicals. Metab Eng.

[25] Jang YS, Kim B, Shin JH, Choi YJ, Choi S, Song CW, Lee J, Park HG, Lee SY (2012). Bio-based production of C2-C6 platform chemicals. Biotechnol Bioeng.

[26] Yim H, Haselbeck R, Niu W, Pujol-Baxley C, Burgard A, Boldt J, Khandurina J, Trawick JD, Osterhout RE, Stephen R, Estadilla J, Teisan S, Schreyer HB, Andrae S, Yang TH, Lee SY, Burk MJ, Van Dien S (2011). Metabolic engineering of *Escherichia coli*for direct production of 1,4-butanediol. Nat Chem Biol.

[27] Xia XX, Qian ZG, Ki CS, Park YH, Kaplan DL, Lee SY (2010). Native-sized recombinant spider silk protein produced in metabolically engineered *Escherichia coli*results in a strong fiber. Proc Natl Acad Sci U S A.

[28] Qian ZG, Xia XX, Lee SY (2009). Metabolic engineering of *Escherichia coli*for the production of putrescine: a four carbon diamine. Biotechnol Bioeng.

[29] Qian ZG, Xia XX, Lee SY (2011). Metabolic engineering of *Escherichia coli*for the production of cadaverine: a five carbon diamine. Biotechnol Bioeng.

[30] Na D, Yoo SM, Chung H, Park H, Park JH, Lee SY (2013). Metabolic engineering of *Escherichia coli*using synthetic small regulatory RNAs. Nat Biotechnol.

[31] Meneguello MO, Mendonca JR, Lancha AH, Costa Rosa LF (2003). Effect of arginine, ornithine and citrulline supplementation upon performance and metabolism of trained rats. Cell Biochem Funct.

[32] Kalinowski J, Bathe B, Bartels D, Bischoff N, Bott M, Burkovski A, Dusch N, Eggeling L, Eikmanns BJ, Gaigalat L, Goesmann A, Hartmann M, Huthmacher K, Kramer R, Linke B, McHardy AC, Meyer F, Mockel B, Pfefferle W, Puhler A, Rey DA, Ruckert C, Rupp O, Sahm H, Wendisch VF, Wiegrabe I, Tauch A (2003). The complete *Corynebacterium glutamicum*ATCC 13032 genome sequence and its impact on the production of L-aspartate-derived amino acids and vitamins. J Biotechnol.

[33] Lu CD (2006). Pathways and regulation of bacterial arginine metabolism and perspectives for obtaining arginine overproducing strains. Appl Microbiol Biotechnol.

[34] Dou WF, Xu MJ, Cai DM, Zhang XM, Rao ZM, Xu ZH (2011). Improvement of L-arginine production by overexpression of a bifunctional ornithine acetyltransferase in *Corynebacterium crenatum*. Appl Biochem Biotechnol.

[35] Glansdorff N, Xu Y, Wendisch V (2007). Microbial Arginine Biosynthesis: Pathway, Regulation and Industrial Production. Amino Acid Biosynthesis - Pathways, Regulation and Metabolic Engineering.

[36] Vyas S, Maas WK (1963). Feedback inhibition of acetylglutamate synthetase by arginine in *Escherichia coli*. Arch Biochem Biophys.

[37] Sakanyan V, Charlier D, Legrain C, Kochikyan A, Mett I, Pierard A, Glansdorff N (1993). Primary structure, partial purification and regulation of key enzymes of the acetyl cycle of arginine biosynthesis in *Bacillus stearothermophilus*: dual function of ornithine acetyltransferase. J Gen Microbiol.

[38] Morizono H, Cabrera-Luque J, Shi DS, Gallegos R, Yamaguchi S, Yu XL, Allewell NM, Malamy MH, Tuchman M (2006). Acetylornithine transcarbamylase: a novel enzyme in arginine biosynthesis. J Bacteriol.

[39] Haas D, Leisinge T, Kurer V (1972). N-acetylglutamate synthetase of *Pseudomonas aeruginosa* - an assay *in vitro*and feedback inhibition by arginine. Eur J Biochem.

[40] Picard FJ, Dillon JR (1989). Cloning and organization of seven arginine biosynthesis genes from *Neisseria gonorrhoeae*. J Bacteriol.

[41] Harris BZ, Singer M (1998). Identification and characterization of the *Myxococcus xanthus argE*gene. J Bacteriol.

[42] Marc F, Weigel P, Legrain C, Almeras Y, Santrot M, Glansdorff N, Sakanyan V (2000). Characterization and kinetic mechanism of mono- and bifunctional ornithine acetyltransferases from thermophilic microorganisms. Eur J Biochem.

[43] Sakanyan V, Kochikyan A, Mett I, Legrain C, Charlier D, Pierard A, Glansdorff N (1992). A reexamination of the pathway for ornithine biosynthesis in a *Thermophilic* and two mesophilic *Bacillus*Species. J Gen Microbiol.

[44] Udaka S (1966). Pathway-specific pattern of control of arginine biosynthesis in bacteria. J Bacteriol.

[45] Martin PR, Mulks MH (1992). Sequence analysis and complementation studies of the *argJ* gene encoding ornithine acetyltransferase from *Neisseria gonorrhoeae*. J Bacteriol.

[46] Hindle Z, Callis R, Dowden S, Rudd BA, Baumberg S (1994). Cloning and expression in *Escherichia coli* of a *Streptomyces coelicolor* A3(2) *argCJB*gene cluster. Microbiology.

[47] Ikeda M, Mitsuhashi S, Tanaka K, Hayashi M (2009). Reengineering of a *Corynebacterium glutamicum*L-arginine and L-citrulline producer. Appl Environ Microbiol.

[48] Xu Y, Labedan B, Glansdorff N (2007). Surprising arginine biosynthesis: a reappraisal of the enzymology and evolution of the pathway in microorganisms. Microbiol Mol Biol Rev.

[49] Yim SH, Jung S, Lee SK, Cheon CI, Song E, Lee SS, Shin J, Lee MS (2011). Purification and characterization of an arginine regulatory protein, ArgR, in *Corynebacterium glutamicum*. J Ind Microbiol Biotechnol.

[50] Schneider J, Wendisch VF (2010). Putrescine production by engineered *Corynebacterium glutamicum*. Appl Microbiol Biotechnol.

[51] Schneider J, Eberhardt D, Wendisch VF (2012). Improving putrescine production by *Corynebacterium glutamicum*by fine-tuning ornithine transcarbamoylase activity using a plasmid addiction system. Appl Microbiol Biotechnol.

[52] Sakanyan V, Petrosyan P, Lecocq M, Boyen A, Legrain C, Demarez M, Hallet JN, Glansdorff N (1996). Genes and enzymes of the acetyl cycle of arginine biosynthesis in *Corynebacterium glutamicum*: enzyme evolution in the early steps of the arginine pathway. Microbiology.

[53] Petri K, Walter F, Persicke M, Ruckert C, Kalinowski J (2013). A novel type of N-acetylglutamate synthase is involved in the first step of arginine biosynthesis in *Corynebacterium glutamicum*. BMC Genomics.

[54] Hwang GH, Cho JY (2010). Identification of a suppressor gene for the arginine-auxotrophic *argJ* mutation in *Corynebacterium glutamicum*. J Ind Microbiol Biotechnol.

[55] Xu Y, Glansdorff N, Labedan B (2006). Bioinformatic analysis of an unusual gene-enzyme relationship in the arginine biosynthetic pathway among marine gamma proteobacteria: implications concerning the formation of N-acetylated intermediates in prokaryotes. BMC Genomics.

[56] Errey JC, Blanchard JS (2005). Functional characterization of a novel ArgA from *Mycobacterium tuberculosis*. J Bacteriol.

[57] Shi D, Sagar V, Jin Z, Yu X, Caldovic L, Morizono H, Allewell NM, Tuchman M (2008). The crystal structure of N-acetyl-L-glutamate synthase from *Neisseria gonorrhoeae*provides insights into mechanisms of catalysis and regulation. J Biol Chem.

[58] Cunin R, Glansdorff N, Pierard A, Stalon V (1986). Biosynthesis and metabolism of arginine in bacteria. Microbiol Rev.

[59] Xu MJ, Rao ZM, Dou WF, Jin J, Xu ZH (2012). Site-directed mutagenesis studies on the L-arginine-binding sites of feedback inhibition in N-Acetyl-l-glutamate Kinase (NAGK) from *Corynebacterium glutamicum*. Curr Microbiol.

[60] Ramon-Maiques S, Fernandez-Murga ML, Gil-Ortiz F, Vagin A, Fita I, Rubio V (2006). Structural bases of feed-back control of arginine biosynthesis, revealed by the structures of two hexameric N-acetylglutamate kinases, from *Thermotoga maritima* and *Pseudomonas aeruginosa*. J Mol Biol.

[61] Theron G, Reid SJ (2011). ArgR-promoter interactions in *Corynebacterium glutamicum*arginine biosynthesis. Biotechnol Appl Biochem.

[62] Lee SY, Cho JY, Lee HJ, Kim YH, Min J (2010). Enhancement of ornithine production in proline-supplemented *Corynebacterium glutamicum*by ornithine cyclodeaminase. J Microbiol Biotechnol.

[63] Hanssler E, Muller T, Jessberger N, Volzke A, Plassmeier J, Kalinowski J, Kramer R, Burkovski A (2007). FarR, a putative regulator of amino acid metabolism in *Corynebacterium glutamicum*. Appl Microbiol Biotechnol.

[64] Lee SY, Park JM, Lee JH, Chang ST, Park JS, Kim YH, Min J (2011). Interaction of transcriptional repressor ArgR with transcriptional regulator FarR at the *argB* promoter region in *Corynebacterium glutamicum*. Appl Environ Microbiol.

[65] Lee SY, Shin HS, Park JS, Kim YH, Min J (2010). Proline reduces the binding of transcriptional regulator ArgR to upstream of *argB* in *Corynebacterium glutamicum*. Appl Microbiol Biotechnol.

[66] Savchenko A, Weigel P, Dimova D, Lecocq M, Sakanyan V (1998). The *Bacillus stearothermophilus argCJBD* operon harbours a strong promoter as evaluated in *Escherichia coli*cells. Gene.

[67] Piette J, Cunin R, Boyen A, Charlier D, Crabeel M, Vanvliet F, Glansdorff N, Squires C, Squires CL (1982). The regulatory region of the divergent *argECBH* operon in *Escherichia coli*K-12. Nucleic Acids Res.

[68] Charlier D, Roovers M, Vanvliet F, Boyen A, Cunin R, Nakamura Y, Glansdorff N, Pierard A (1992). Arginine regulon of *Escherichia coli* K-12: A study of repressor operator interactions and of *in vitro* binding affinities versus *in vivo*repression. J Mol Biol.

[69] Bringel F, Frey L, Boivin S, Hubert JC (1997). Arginine biosynthesis and regulation in *Lactobacillus plantarum*: the *carA* gene and the *argCJBDF*cluster are divergently transcribed. J Bacteriol.

[70] RodriguezGarcia A, Ludovice M, Martin JF, Liras P (1997). Arginine boxes and the *argR* gene in *Streptomyces clavuligerus*: evidence for a clear regulation of the arginine pathway. Mol Microbiol.

[71] Walker JB (1955). Canavanine and homoarginine as antimetabolites of arginine and lysine in yeast and algae. J Biol Chem.

[72] Nakayama K, Yoshida H (1972). Fermentative production of L-arginine. Agric Biol Chem.

[73] Yoshida H, Araki K, Nakayama K (1981). Fermentative production of L-arginine. 5. L-Arginine production by arginine analog-resistant mutants of microorganisms. Agric Biol Chem.

[74] Kato J, Kisumi M, Takagi T, Chibata I (1977). Increase in arginine and citrulline production by 6-azauracil-resistant mutants of *Bacillus subtilis*. Appl Environ Microbiol.

[75] Kubota K, Onoda T, Kamijo H, Yoshinag F, Okumura S (1973). Microbial production of L-arginine. 1. Production of L-arginine by mutants of glutamic acid-producing bacteria. J Gen Appl Microbiol.

[76] Xu H, Dou WF, Xu HY, Zhang XM, Rao ZM, Shi ZP, Xu ZH (2009). A two-stage oxygen supply strategy for enhanced L-arginine production by *Corynebacterium crenatum*based on metabolic fluxes analysis. Biochem Eng J.

[77] Xu MJ, Rao ZM, Xu H, Lan CY, Dou WF, Zhang XM, Xu HY, Jin JA, Xu ZH (2011). Enhanced production of L-arginine by expression of *Vitreoscilla* hemoglobin using a novel expression system in *Corynebacterium crenatum*. Appl Biochem Biotechnol.

[78] Xu MJ, Rao ZM, Yang J, Xia HF, Dou WF, Jin J, Xu ZH (2012). Heterologous and homologous expression of the arginine biosynthetic *argC* ~ *H* cluster from *Corynebacterium crenatum*for improvement of L-arginine production. J Ind Microbiol Biotechnol.

[79] Choi DK, Ryu WS, Choi CY, Park YH (1996). Production of L-ornithine by arginine auxotrophic mutants of *Brevibacterium ketoglutamicum*in dual substrate-limited continuous culture. J Ferment Bioeng.

[80] Hwang JH, Hwang GH, Cho JY (2008). Effect of increased glutamate availability on L-ornithine production in *Corynebacterium glutamicum*. J Microbiol Biotechnol.

[81] Schneider J, Niermann K, Wendisch VF (2011). Production of the amino acids L-glutamate, L-lysine, L-ornithine and L-arginine from arabinose by recombinant *Corynebacterium glutamicum*. J Biotechnol.

[82] Lu DM, Liu JZ, Mao ZW (2012). Engineering of *Corynebacterium glutamicum*to enhance L-ornithine production by gene knockout and comparative proteomic analysis. Chin J Chem Eng.

[83] Hwang GH, Cho JY (2012). Implication of gluconate kinase activity in L-ornithine biosynthesis in *Corynebacterium glutamicum*. J Ind Microbiol Biotechnol.

[84] Jiang LY, Zhang YY, Li Z, Liu JZ (2013). Metabolic engineering of *Corynebacterium glutamicum*for increasing the production of L-ornithine by increasing NADPH availability. J Ind Microbiol Biotechnol.

[85] Jiang LY, Chen SG, Zhang YY, Liu JZ (2013). Metabolic evolution of *Corynebacterium glutamicum*for increased production of L-ornithine. BMC Biotechnol.

[86] Kim SY, Lee J, Lee SY: Metabolic engineering of *Corynebacterium glutamicum* for the production of L-ornithine. *Biotechnol Bioeng.* in press., 10.1002/bit.25440 25163446

[87] Meiswinkel T, Rittmann D, Lindner SN, Wendisch VF (2013). Crude glycerol-based production of amino acids and putrescine by *Corynebacterium glutamicum*. Bioresour Technol.

[88] Aboulmagd E, Voss I, Oppermann-Sanio FB, Steinbuchel A (2001). Heterologous expression of cyanophycin synthetase and cyanophycin synthesis in the industrial relevant bacteria *Corynebacterium glutamicum* and *Ralstonia eutropha* and in *Pseudomonas putida*. Biomacromolecules.

[89] Frey KM, Oppermann-Sanio FB, Schmidt H, Steinbuchel A (2002). Technical-scale production of cyanophycin with recombinant strains of *Escherichia coli*. Appl Environ Microbiol.

[90] Voss I, Diniz SC, Aboulmagd E, Steinbuchel A (2004). Identification of the *Anabaena* sp strain PCC7120 cyanophycin synthetase as suitable enzyme for production of cyanophycin in gram-negative bacteria like *Pseudomonas putida* and *Ralstonia eutropha*. Biomacromolecules.

[91] Elbahloul Y, Krehenbrink M, Reichelt R, Steinbuchel A (2005). Physiological conditions conducive to high cyanophycin content in biomass of *Acinetobacter calcoaceticus*strain ADP1. Appl Environ Microbiol.

[92] Voss I, Steinbuchel A (2006). Application of a KDPG-aldolase gene-dependent addiction system for enhanced production of cyanophycin in *Ralstonia eutropha*strain H16. Metab Eng.

[93] Kroll J, Klinter S, Steinbuchel A (2011). A novel plasmid addiction system for large-scale production of cyanophycin in *Escherichia coli*using mineral salts medium. Appl Microbiol Biotechnol.

[94] Lin KC, Elbahloul Y, Steinbuchel A (2012). Physiological conditions conducive to high cell density and high cyanophycin content in *Ralstonia eutropha*strain H16 possessing a KDPG aldolase gene-dependent addiction system. Appl Microbiol Biotechnol.

[95] Kind S, Jeong WK, Schroder H, Zelder O, Wittmann C (2010). Identification and elimination of the competing N-acetyldiaminopentane pathway for improved production of diaminopentane by *Corynebacterium glutamicum*. Appl Environ Microbiol.

[96] Kroll J, Klinter S, Schneider C, Voss I, Steinbuchel A (2010). Plasmid addiction systems: perspectives and applications in biotechnology. Microb Biotechnol.

[97] Borzi A (1886). Malpighia. Le comunicazioni intracellulari delle Nostochinee.

[98] Schwamborn M (1998). Chemical synthesis of polyaspartates: a biodegradable alternative to currently used polycarboxylate homo- and copolymers. Polym Degrad Stab.

[99] Zotz RJ, Schenk S, Kuhn A, Schlunken S, Krone V, Bruns W, Genth S, Schuler G (2001). Safety and efficacy of LK565 - a new polymer ultrasound contrast agent. Z Kardiol.

[100] Lutte S, Pohlmann A, Zaychikov E, Schwartz E, Becher JR, Heumann H, Friedrich B (2012). Autotrophic production of stable-isotope-labeled arginine in *Ralstonia eutropha*Strain H16. Appl Environ Microbiol.

[101] Elbahloul Y, Steinbuchel A (2006). Engineering the genotype of *Acinetobacter*sp strain ADP1 to enhance biosynthesis of cyanophycin. Appl Environ Microbiol.

[102] Kroll J, Steinle A, Reichelt R, Ewering C, Steinbuchel A (2009). Establishment of a novel anabolism-based addiction system with an artificially introduced mevalonate pathway: complete stabilization of plasmids as universal application in white biotechnology. Metab Eng.

