# Supplementary figures and images for: Metabolic engineering of microorganisms for the production of L-arginine and its derivatives
Source: Microb Cell Fact. 2014 Dec 3;13:166. doi: 10.1186/s12934-014-0166-4 (PMC4258820; doi:10.1186/s12934-014-0166-4)

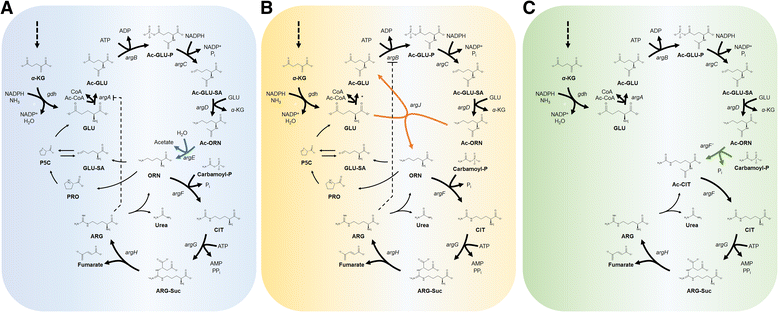

Supplement: Supplementary file 1 — Authors’ original file for figure 1 [file 12934_2014_166_MOESM1_ESM.gif]

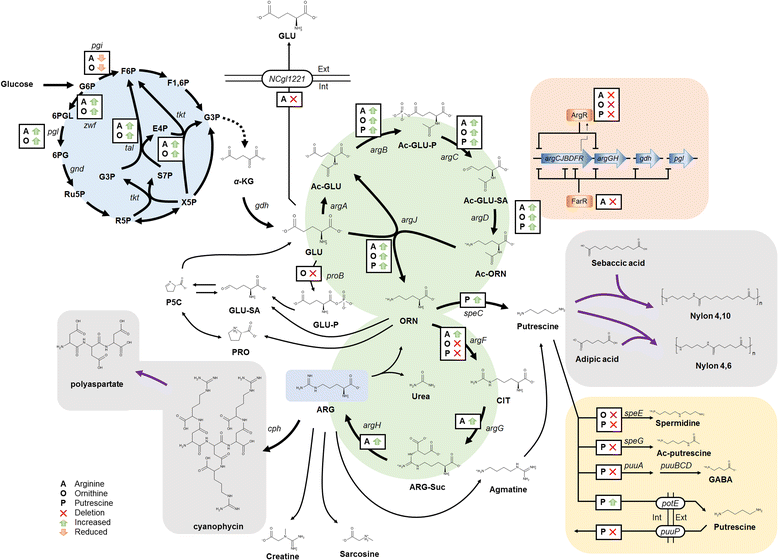

Supplement: Supplementary file 2 — Authors’ original file for figure 2 [file 12934_2014_166_MOESM2_ESM.gif]
